# Association of Preoperative Basal Inflammatory State, Measured by Plasma suPAR Levels, with Intraoperative Sublingual Microvascular Perfusion in Patients Undergoing Major Non-Cardiac Surgery

**DOI:** 10.3390/jcm11123326

**Published:** 2022-06-10

**Authors:** Athanasios Chalkias, Nikolaos Papagiannakis, Bernd Saugel, Moritz Flick, Konstantina Kolonia, Zacharoula Angelopoulou, Dimitrios Ragias, Dimitra Papaspyrou, Aikaterini Bouzia, Nicoletta Ntalarizou, Konstantinos Stamoulis, Aikaterini Kyriakaki, Jesper Eugen-Olsen, Eleni Laou, Eleni Arnaoutoglou

**Affiliations:** 1 Department of Anesthesiology, Faculty of Medicine, University of Thessaly, 41500 Larisa, Greece; kon.kolonia@gmail.com (K.K.); zacharula@gmail.com (Z.A.); mjr9898@hotmail.gr (D.R.); livkristine1991@hotmail.com (D.P.); catbouzia@yahoo.gr (A.B.); nntalarizou@gmail.com (N.N.); konstaarist@windowslive.com (K.S.); katrinkyr@hotmail.com (A.K.); elenilaou1@gmail.com (E.L.); earnaout@gmail.com (E.A.); 2 Outcomes Research Consortium, Cleveland, OH 44195, USA; bernd.saugel@gmx.de; 3 Hellenic Society of Cardiopulmonary Resuscitation, 10434 Athens, Greece; 4 First Department of Neurology, Eginition University Hospital, Medical School, National and Kapodistrian University of Athens, 11528 Athens, Greece; nikolas.papagia@gmail.com; 5 Department of Anesthesiology, Center of Anesthesiology and Intensive Care Medicine, University Medical Center Hamburg-Eppendorf, 20251 Hamburg, Germany; m.flick@uke.de; 6 Department of Clinical Research, Copenhagen University Hospital Hvidovre, 2650 Hvidovre, Denmark; jespereugenolsen@gmail.com

**Keywords:** anesthesiology, inflammation, suPAR, microcirculation, surgery, perioperative

## Abstract

It remains unknown whether chronic systemic inflammation is associated with impaired microvascular perfusion during surgery. We evaluated the association between the preoperative basal inflammatory state, measured by plasma soluble urokinase-type plasminogen activator receptor (suPAR) levels, and intraoperative sublingual microcirculatory variables in patients undergoing major non-cardiac surgery. Plasma suPAR levels were determined in 100 non-cardiac surgery patients using the suPARnostic^®^ quick triage lateral flow assay. We assessed sublingual microcirculation before surgical incision and every 30 min during surgery using Sidestream Darkfield (SDF+) imaging and determined the De Backer score, the Consensus Proportion of Perfused Vessels (Consensus PPV), and the Consensus PPV (small). Elevated suPAR levels were associated with lower intraoperative De Backer score, Consensus PPV, and Consensus PPV (small). For each ng mL^−1^ increase in suPAR, De Backer score, Consensus PPV, and Consensus PPV (small) decreased by 0.7 mm^−1^, 2.5%, and 2.8%, respectively, compared to baseline. In contrast, CRP was not significantly correlated with De Backer score (r = −0.034, *p* = 0.36), Consensus PPV (r = −0.014, *p* = 0.72) or Consensus PPV Small (r = −0.037, *p* = 0.32). Postoperative De Backer score did not change significantly from baseline (5.95 ± 3.21 vs. 5.89 ± 3.36, *p* = 0.404), while postoperative Consensus PPV (83.49 ± 11.5 vs. 81.15 ± 11.8, *p* < 0.001) and Consensus PPV (small) (80.87 ± 13.4 vs. 78.72 ± 13, *p* < 0.001) decreased significantly from baseline. In conclusion, elevated preoperative suPAR levels were associated with intraoperative impairment of sublingual microvascular perfusion in patients undergoing elective major non-cardiac surgery.

## 1. Introduction

Chronic systemic inflammation refers to persistent, low-grade inflammation and is involved in the pathogenesis of a wide variety of chronic non-communicable diseases. Despite the established link between inflammation and organ injury, the underlying pathophysiology is not well elucidated. During inflammation, however, the microvasculature exhibits characteristic phenotypic changes that isolate the pathological region from healthy tissue and the systemic circulation [1]. Indeed, inflammation impairs microcirculatory vasomotor function, decreases capillary perfusion and vascular integrity, increases adhesion of leukocytes and platelets, activates the coagulation cascade, and increases the rate of proliferation of blood and lymphatic vessels [2,3,4,5,6]. In turn, the inflammation-induced microvascular changes may enhance and sustain the inflammatory response [1,7]. From a physiological perspective, impaired microcirculatory flow and chronic inflammation can provoke organ dysfunction and complications and worsen the outcome after surgery [8,9].

Currently, there are no biomarkers that can be routinely used in the prediction of outcomes and advanced planning in the perioperative setting [10,11]. The soluble urokinase-type plasminogen activator receptor (suPAR) is a marker of chronic systemic inflammation [5]. Healthy individuals can also be genetically predisposed to higher or lower suPAR levels, which gives suPAR a potential prognostic value in associated diseases [6]. Of note, mounting evidence from studies including cancer and non-cancer patients suggests that suPAR can serve as an independent predictor of postoperative complications and outcomes compared to other biomarkers [12,13,14,15,16,17,18,19,20,21]. This could be explained in part by the stability of suPAR, which is significantly higher than that of other markers (e.g., C-reactive protein (CRP) or proinflammatory cytokines) and remains unaffected by general anesthesia and operative trauma [22]. Interestingly, plasma suPAR level is an independent predictor of coronary microvascular function, with a doubling in suPAR being associated with a 30% decrease in microcirculatory flow reserve [23]. Furthermore, suPAR is implicated in systemic sclerosis-related microvascular abnormalities [24].

We hypothesized that chronic systemic inflammation (basal inflammatory state) can compromise intraoperative microvascular perfusion and affect the outcome of patients undergoing non-cardiac surgery. To test our hypothesis, we evaluated the association between preoperative suPAR levels and intraoperative sublingual microvascular perfusion in patients undergoing elective major non-cardiac surgery.

## 2. Materials and Methods

### 2.1. Design

This was a prospective observational study conducted in the University Hospital of Larisa, Greece, from February 2019 to September 2020. Ethical approval for this study was provided by the Ethics Committee of the Hospital on 11 December 2018 (No. 60580). The study was designed in accordance with the declaration of Helsinki and was registered at ClinicalTrials.gov (NCT03851965). Written informed consent was obtained from all patients or next-of-kin.

### 2.2. Study Objectives

Our goal was to investigate the association between preoperative plasma suPAR, reflecting the basal inflammatory state, and intraoperative sublingual microcirculation.

### 2.3. Patient Eligibility

Consecutive adults who were scheduled to undergo elective major non-cardiac surgery with an expected duration of ≥2 h, under general anesthesia, were eligible for inclusion. Patients were American Society of Anesthesiologists (ASA) physical status I to IV. All operative approaches were eligible, including open and laparoscopic procedures.

We excluded patients with any infection within the previous month; severe liver disease; need for renal replacement therapy; allergies; inflammatory or immune disorders; asthma; obesity (BMI ≥ 30 kg m^−2^); mental disability or severe psychiatric disease; alcohol abuse; connective tissue disease including rheumatoid arthritis, ankylosing spondylitis, and systemic lupus erythematosus. We also excluded patients who had previously received an organ transplant; who were treated with steroids, antipsychotic or anti-inflammatory/immunomodulatory medication within the previous three months or with opioids during the past week; and who were involved in another study.

### 2.4. Anesthetic Management

Before anesthesia induction, patients were given 5 mL kg^−1^ of a balanced crystalloid solution to compensate for preoperative fasting and anesthetic-induced vasodilation. Anesthesia was induced in the supine position and included midazolam 0.15–0.35 mg kg^−1^ over 20–30 s, fentanyl 1 μg kg^−1^, ketamine 0.2 mg kg^−1^, propofol 1.5–2 mg kg^−1^, rocuronium 0.6 mg kg^−1^, and a fraction of inspired oxygen of 0.7 [25,26]. After tracheal intubation, patients were mechanically ventilated using a lung-protective strategy with tidal volume of 7 mL kg^−1^, positive end-expiratory pressure of 6–8 cmH_2_O, and plateau pressure <30 cm H_2_O (Draeger Perseus A500; Drägerwerk AG and Co., Lübeck, Germany).

General anesthesia was maintained by inhalation of desflurane at an initial 1.0 minimal alveolar concentration. Thereafter, the depth of anesthesia was adjusted to maintain Bispectral Index (BIS, Covidien, Paris, France) between 40 and 60 [27,28]. Intraoperative inspired oxygen was then adjusted to maintain an arterial oxygen partial pressure of 80–100 mmHg, and normocapnia was maintained by adjusting the respiratory rate as needed [29,30,31]. Normothermia (37 °C) and normoglycemia were maintained during the perioperative period.

Throughout surgery, balanced crystalloids were given at a rate of 2 mL kg^−1^ h^−1^. Surgery-related blood losses were compensated by infusing balanced crystalloids (2:1 ratio) or 6% hydroxyethyl starch 130/0.4 (1:1 ratio). Packed red cells were transfused when the hemoglobin level dropped below 9–10 g dL^−1^ in patients with cardiovascular comorbidities and the elderly, or below 8 g dL^−1^ in those without cardiac comorbidities.

### 2.5. Sampling and Laboratory Measurements

Venous blood was sampled immediately after arrival to the operating room before induction of anesthesia. Blood samples drawn from all patients were collected in EDTA tubes and were centrifuged at 3000× *g* for 1 min. Plasma suPAR levels were determined using the suPARnostic^®^ Quick Triage lateral flow assay (ViroGates, Birkerød, Denmark). This is an easy-to-use quantitative test that is based on the lateral flow principle. The test consists of a nitrocellulose membrane with two immobilized antibody zones and a running buffer. The quantitative results were read within 20 min by an optical aLF Reader (Qiagen, Hilden, Germany). We considered suPAR levels higher than 5.5 ng mL^−1^ as a clinically important indicator of systemic inflammation, as previously suggested [32]. A second cut-off at 10 ng mL^−1^ was chosen to indicate especially high suPAR levels [12].

### 2.6. Sublingual Microcirculation Analysis

Sublingual microcirculation was monitored using sidestream dark field (SDF+) imaging (Microscan; Microvision Medical BV, Amsterdam, The Netherlands) [33]. Microcirculation was assessed 30 min after induction of general anesthesia before surgical incision and then every 30 min until emergence from anesthesia. At each measurement point, we recorded sublingual microcirculation videos from at least five sites. All videos were recorded by the same investigator who was blinded to patient data and suPAR measurements. To optimize video quality, we tried to avoid pressure and movement artefacts, optimized focus and illumination, and cleaned saliva and/or blood from the sublingual mucosa. Before analysis, all sublingual perfusion videos were evaluated by two experienced raters blinded to all patient data, according to a modified microcirculation image quality score (MIQS) [34]. The best three videos from each recording were analyzed offline by a blinded investigator with the AVA4.3C Research Software (Microvision Medical, Amsterdam, the Netherlands) [35,36,37,38,39,40]. We analyzed the De Backer score as density score and the Consensus Proportion of Perfused Vessels (Consensus PPV) and Consensus PPV (small) as flow scores.

### 2.7. Data Collection, Monitoring, and Management

Data analysis was based on predefined and contemporaneously recorded measurements. Data collection included demographics, ASA physical status, Modified Frailty Index, POSSUM risk score, anesthesia variables, general blood count, biochemistry profile, suPAR, and CRP. We also used the Clavien–Dindo Classification and the Comprehensive Complication Index (CCI) to assess postoperative complications, morbidity, and mortality in our patients [41,42]. Remote monitoring was performed to signal early aberrant patterns, issues with consistency, credibility, and other anomalies. Any missing and outlier data values were individually revised and completed or corrected whenever possible. This work is reported according to STROCSS criteria [43].

### 2.8. Statistical Analysis

Statistical analysis was performed using R v.4.0.0 (R core team, www.r-project.org). Descriptive data are shown as median with interquartile range (IQR). Spearman’s correlation coefficient was used to compare microcirculation variables with hemodynamic and clinical characteristics and with morbidity and mortality scores. The Benjamini–Hochberg false discovery rate correction was applied in the resulting *p*-values to account for the multiple numbers of tests. Adjusted *p*-values less than 0.05 were deemed significant. Linear mixed effects (LME) models with Restricted Maximum Likelihood Estimation were used in order to assess the different effects in microcirculatory variables. LME was used instead of repeated-measures ANOVA due to the different intra-operative duration between patients and the associated missing values. Three linear mixed models were constructed, for each one of the microcirculatory variables (De Backer score, Consensus PPV, and Consensus PPV (small)), with suPAR and the different time points as fixed factors and the different participants as random factors. Changes in intraoperative sublingual microcirculation variables were assessed by the second term of the model. We could not identify previous research investigating the association of suPAR with intraoperative sublingual microcirculation. As no data exist, we chose to include 100 individuals because we expected that this number could reveal important associations and generate results to be used for sample size estimation in future large-scale studies.

## 3. Results

Demographic and clinical characteristics of patients are depicted in Table 1 and Table S1.

**Figure 1:**
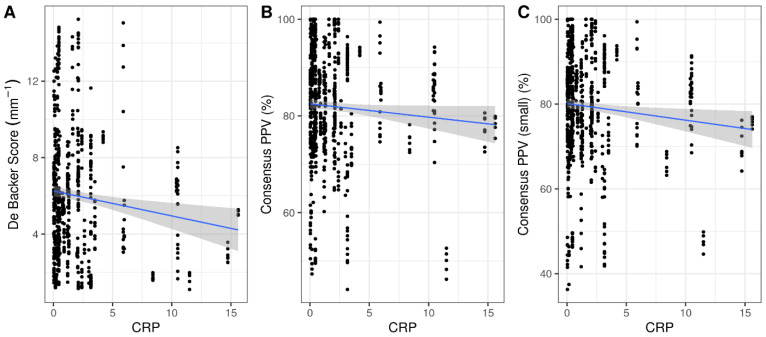
Correlation of preoperative CRP with De Backer score (mm^−1^) (**A**), Consensus PPV (%) (**B**), and Consensus PPV (small) (%) (**C**). PPV, Proportion of Perfused Vessels.

Elevated levels of preoperative suPAR were associated with a decrease in all intraoperative sublingual microcirculation variables. Specifically, we observed a decrease of 0.7 mm^−1^ in the De Backer score, a decrease of 2.5% in the Consensus PPV, and a decrease of 2.8% in the Consensus PPV (small) from baseline measurement for each ng mL^−1^ increase in suPAR (Table 2). In contrast, CRP was not significantly correlated with De Backer score (r = −0.034, *p* = 0.36), Consensus PPV (r = −0.014, *p* = 0.72) or Consensus PPV Small (r = −0.037, *p* = 0.32) (Figure 1).

**Figure 2:**
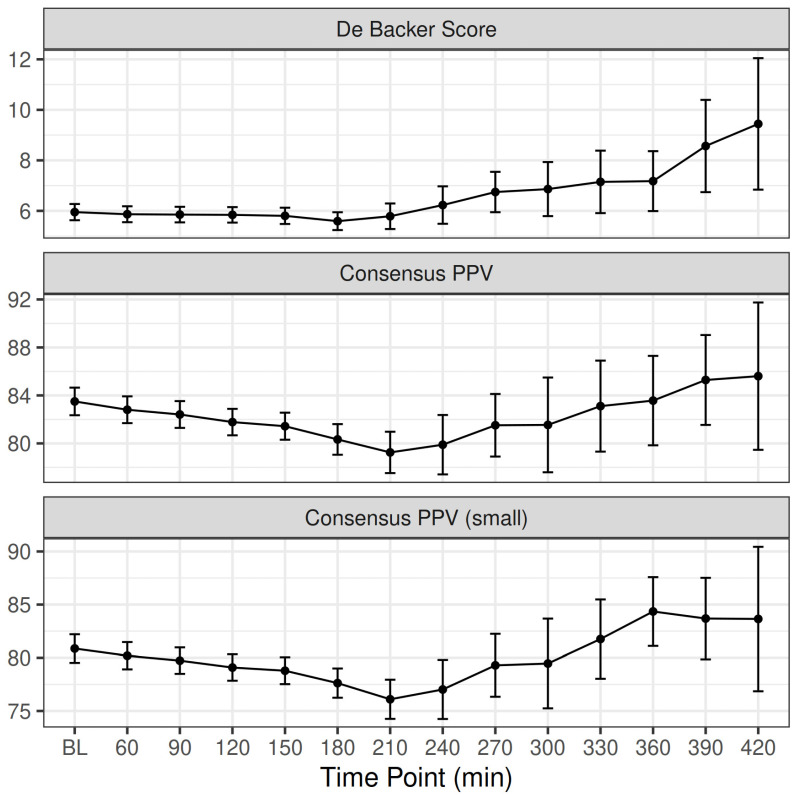
Intraoperative change in De Backer score (mm^−1^), Consensus PPV (%), and Consensus PPV (small) (%) with time. BL, baseline measurement (30 min). Number of patients at each time point: 30–120 min: 100 patients; 150 min: 96 patients; 180 min: 72 patients; 210 min: 49 patients; 240 min; 27 patients; 270 min: 22 patients; 300 min: 12 patients; 330 min: 8 patients; 360 min: 8 patients: 390 min: 4 patients; 420 min: 3 patients.

Figure 2 depicts the estimated differences between different time points and baseline (30 min). The postoperative De Backer score did not change significantly from baseline (5.95 ± 3.21 mm^−1^ vs. 5.89 ± 3.36 mm^−1^, *p* = 0.404), while postoperative Consensus PPV (83.49 ± 11.5% vs. 81.15 ± 11.8%, *p* < 0.001) and Consensus PPV (small) (80.87 ± 13.4% vs. 78.72 ± 13%, *p* < 0.001) decreased significantly from baseline. Patients with lower preoperative suPAR levels showed a tendency to restore DeBacker Score (r = −0.148, *p* = 0.036) after the end of surgery and before emergence from anesthesia, but not Consensus PPV (r = −0.084, *p* = 0.238) or Consensus PPV Small (r = −0.013, *p* = 0.853).

Baseline suPAR levels were significantly correlated with CCI (r = 0.479; *p* < 0.001). Additionally, baseline suPAR levels were correlated with age, urea, and creatinine levels (r = 0.276, *p* = 0.005; r = 0.220, *p* = 0.027; r = 0.248, and *p* = 0.013, respectively). The De Backer score, the Consensus PPV, and the Consensus PPV (small) correlated with CCI and POSSUM risk score (Table 3 and Table S2). Only 19 out of 100 patients received 6% hydroxyethyl starch 130/0.4, with no apparent association with the microcirculatory variables (r = −0.054, *p* = 0.15).

## 4. Discussion

In this observational study with patients undergoing elective major non-cardiac surgery, we observed a statistically significant decrease of 0.7 mm^−1^ in the De Backer score, 2.5% in the Consensus PPV, and 2.8% in the Consensus PPV (small) from baseline measurement for each ng mL^−1^ increase in preoperative suPAR level. Preoperative CPR levels were not correlated with intraoperative sublingual microcirculation. Our findings suggest that basal inflammatory state, measured by suPAR, synergizes with operative trauma to mediate microvascular dysfunction, which in turn has been suggested to mediate postoperative complications [44]. The association between the level of preoperative systemic inflammation and intraoperative impairment of sublingual microvascular perfusion may be particularly important during prolonged anesthetic/surgical duration.

Chronic systemic inflammation is a major problem in the general population [45]. Its level is typically assessed using biomarkers of acute inflammation, such as CRP, white blood cell count, or proinflammatory cytokines. However, these markers are rapidly up- and down-regulated, making their quantification time-sensitive. In recent years, suPAR has been described as a more stable biomarker of chronic inflammation and was closely linked to cognitive impairment and development of inflammation-related diseases [46,47]. In addition, higher suPAR levels reduce the function of the urokinase plasminogen activator receptor system in endothelial cells and may thus lead to microcirculatory flow abnormalities [24]. Considering that both the expression and release of suPAR are upregulated by immune activation, we hypothesized that the intraoperative impairment of microcirculatory flow correlates with the preoperative levels of suPAR. In the present study, we observed a decrease in the De Backer score, Consensus PPV, and Consensus PPV (small) for each ng mL^−1^ increase in preoperative suPAR. In contrast, preoperative CPR levels were not correlated with intraoperative impairment of sublingual microcirculation. To the best of our knowledge, this is the first report of the inverse correlation between chronic systemic inflammation, reflected by preoperative suPAR levels, and sublingual microcirculatory variables in perioperative medicine. Interestingly, evidence from other populations shows that increasing suPAR levels are associated with a more severe microvascular involvement [23,24], but none of these studies investigated sublingual microcirculation. Although other biomarkers of endothelial cell activation and injury or glycocalyx injury may also increase intraoperatively (indicating the magnitude of endothelial damage), only suPAR, to our knowledge, exerts proinflammatory functions [46] and can intensify the effects of surgical stress, further impairing microcirculatory flow. Furthermore, general anesthesia and operative trauma do not influence the perioperative suPAR levels despite the activation of systemic inflammatory reaction [22]. Therefore, preoperative suPAR may be a promising biomarker for assessing the effects of chronic systemic inflammation on perioperative microcirculation and organ perfusion.

The evidence suggesting an association between microvascular disease and poor outcomes after non-cardiac surgery is scarce. In a prospective observational study with 25 patients receiving standard perioperative care following major abdominal surgery, microcirculatory flow impairment was associated with higher rates of postoperative complications [48]. In addition, a recent systematic review and meta-analysis reported significant sublingual microcirculatory flow alterations during the immediate and early postoperative period [44]. However, the exact underlying mechanisms between chronic inflammation and microvascular impairment are not fully understood. The induction of an inflammatory response can impair the ability of the microvasculature to coordinate a vasodilatory response in different organs, especially in hypertensive individuals [2]. Furthermore, suPAR, hypoxia, and ischemia can all impair vasodilation [3,4], while a diminished microcirculatory blood flow may in turn enhance the inflammatory response [1,7], forming a dangerous vicious cycle. The crucial point for this seems to be the change of the endothelium from a quiescent into an active state. In patients with chronic inflammation, the endothelium has been activated much earlier than the time of surgery and is associated with elevated suPAR levels [23,36]. In these individuals, graded changes in vascular shear rate, even for brief periods, elicit progressive recruitment of both rolling and firmly adherent leukocytes [5]. The activated leukocytes cause oxidative and enzymatic degradation of the glycocalyx and further impair microvascular perfusion [6,44]. All these are consistent with our findings, which indicate a strong association between suPAR, microcirculatory flow impairment, and postoperative complications. Given that sublingual microcirculatory alterations occurring during surgery may be maintained during the immediate and early postoperative period [44,48], the role of chronic inflammation appears to be important in the development of postoperative complications.

This study has several limitations. It is a single-center study, and therefore, the results may not be generalizable. Apart from suPAR, we investigated the association between CRP and sublingual microcirculation. We focused on suPAR based on the evidence suggesting that it can better serve as an independent prognostic marker of postoperative complications and outcomes compared to other standard markers, such as CRP, and because it is not affected by anesthesia and operative trauma [12,13,14,15,16,17,18,19,20,21,22]. Furthermore, we assessed the microcirculation using the AVA 4.3C software, which may not be sufficiently validated at this time. However, all the currently used software packages have limitations [36]. Of note, the AVA4.3C has been used in studies published in journals specializing in microcirculation and in other peer-reviewed journals [37,38,39,40,49,50,51], while at least three published studies report that the AVA4.3C is a validated automatic algorithm-software [37,38,39]. Furthermore, we analyzed several videos at each time, used continuous measurements, which allowed a better comparison over time, and followed the recommended methodology for sublingual microcirculation videos [36]. Another limitation is that we had many exclusion criteria to minimize the effects of other diseases, conditions, or medication on microcirculatory blood flow. As there are no data on the association between preoperative suPAR and intraoperative microcirculation, we chose to include 100 individuals in this study. The encouraging results of the present analysis can be used for sample size estimation in future large-scale studies. In addition, we did not assess other biomarkers of endothelial cell activation and injury or glycocalyx injury. Although anesthetics may affect microcirculatory flow, we used desflurane for maintenance because it produces stable effects on the microcirculation [40]. Despite these limitations, the present study is the largest prospective cohort investigating the association between chronic systemic inflammation and sublingual microcirculatory perfusion in perioperative medicine.

## 5. Conclusions

Elevated preoperative suPAR levels are associated with decreased intraoperative De Backer score, Consensus PPV, and Consensus PPV (small) in patients undergoing elective major non-cardiac surgery. These findings may help in identifying patients at risk who may benefit from advanced perioperative care planning.

## Figures and Tables

**Table 1:** Demographic and clinical characteristics of patients undergoing major non-cardiac surgery.

Age, years (mean ± SD)		67.2 ± 12.5
Sex (Male), n (%)		68 (68%)
ASA physical status, n (%)	II	17 (17%)
	III	43 (43%)
	IV	40 (40%)
Type of surgery, n (%)	Endocrinological	1 (1%)
	Gastrointestinal	42 (42%)
	Gastrointestinal/Gynecological	1 (1%)
	Gynecological	5 (5%)
	Thoracic	1 (1%)
	Urological	16 (16%)
	Vascular	32 (32%)
	Various	2 (2%)
Medication		
Aspirin, n (%)	No	74 (74%)
	Yes	26 (26%)
Beta blocker, n (%)	No	64 (64%)
	Yes	36 (36%)
ACEi, n (%)	No	86 (86%)
	Yes	14 (14%)
Diuretic, n (%)	No	78 (78%)
	Yes	22 (22%)
Comorbidities		
Ischemic heart disease, n (%)	No	78 (78%)
	Yes	22 (22%)
Arterial hypertension, n (%)	No	36 (36%)
	Yes	64 (64%)
Hypercholesterolemia, n (%)	No	51 (51%)
	Yes	49 (49%)
Diabetes, n (%)	No	86 (86%)
	Yes	14 (14%)
Stroke, n (%)	No	93 (93%)
	Yes	7 (7%)
COPD, n (%)	No	76 (76%)
	Yes	24 (24%)
Asthma, n (%)	No	98 (98%)
	Yes	2 (2%)
Other, n (%)	No	37 (37%)
	Yes	63 (63%)
suPAR (ng mL^−1^), mean ± SD		8.09 ± 3.69

ASA, American Society of Anesthesiologists; ACEi, angiotensin-converting enzyme inhibitors; COPD, chronic obstructive pulmonary disease.

**Table 2:** Association of suPAR with intraoperative sublingual microcirculatory perfusion.

	Beta Coefficient *	Standard Error	*p*-Value
De Backer score	−0.716	0.041	<0.001
Consensus PPV	−2.490	0.159	<0.001
Consensus PPV (small)	−2.835	0.1713	<0.001

* Represents the increase/decrease in the respective microcirculatory variable for each ng mL^−1^ increase in suPAR. PPV, Proportion of Perfused Vessels.

**Table 3:** Correlation of microcirculation variables with morbidity and mortality scores.

**Preoperatively—30 min after Induction of General Anesthesia (Before Surgical Incision)**			
		**Spearman’s Rho**	**Adjusted *p*-Value**
De Backer score	Modified Frailty Index	−0.156	0.12
	Comprehensive Complication Index	−0.251	0.012
Consensus PPV (%)	Modified Frailty Index	−0.102	0.315
	Comprehensive Complication Index	−0.28	0.005
Consensus PPV (small) (%)	Modified Frailty Index	−0.075	0.456
	Comprehensive Complication Index	−0.277	0.005
**Postoperatively—before Emergence from Anesthesia**			
		**Spearman’s Rho**	**Adjusted *p*-Value**
De Backer score	Modified Frailty Index	−0.184	0.067
	Comprehensive Complication Index	−0.289	0.003
Consensus PPV (%)	Modified Frailty Index	−0.221	0.027
	Comprehensive Complication Index	−0.328	0.001
Consensus PPV (small) (%)	Modified Frailty Index	−0.189	0.06
	Comprehensive Complication Index	−0.327	0.001

PPV, Proportion of Perfused Vessels.

## Data Availability

Data can be made available upon request after publication through a collaborative process. Researchers should provide a methodically sound proposal with specific objectives in an approval proposal. Please contact the corresponding author for additional information.

## Supplementary Materials

The following supporting information can be downloaded at: https://www.mdpi.com/article/10.3390/jcm11123326/s1, Table S1: Preoperative and postoperative arterial blood gases. Table S2: Demographics, surgical procedures, and postoperative complications.
