# Supplementary material for: Association of Preoperative Basal Inflammatory State, Measured by Plasma suPAR Levels, with Intraoperative Sublingual Microvascular Perfusion in Patients Undergoing Major Non-Cardiac Surgery
Source: J Clin Med. 2022 Jun 10;11(12):3326. doi: 10.3390/jcm11123326 (PMC9225100; doi:10.3390/jcm11123326)
Supplement: Supplementary file 1 [file jcm-11-03326-s001.zip › Table S1.pdf]

Table S1. Preoperative and postoperative arterial blood gases

	Preoperatively	Postoperatively	Adjusted p-values
pH	7.38 (0.02)	7.40 (0.03)	<0.001
$PaCO_2$ (mmHg), mean (SD)	38.22 (3.3)	39.88 (2.6)	<0.001
HCO_3 (mmol/L), mean (SD)	23.72 (2.5)	25.5 (1.8)	<0.001
Lactate (mmol/L), mean (SD)	1.25 (0.4)	0.86 (0.2)	<0.001
SaO_2 (%), mean (SD)	97.56 (1.8)	99.81 (0.6)	<0.001
SpO_2 (%), mean (SD)	97.09 (1.9)	98.86 (1.2)	<0.001
