# Supplementary material for: Association of Preoperative Basal Inflammatory State, Measured by Plasma suPAR Levels, with Intraoperative Sublingual Microvascular Perfusion in Patients Undergoing Major Non-Cardiac Surgery
Source: J Clin Med. 2022 Jun 10;11(12):3326. doi: 10.3390/jcm11123326 (PMC9225100; doi:10.3390/jcm11123326)
Supplement: Supplementary file 1 [file jcm-11-03326-s001.zip › Table S2.pdf]

Table S2. Demographics, surgical procedures, and postoperative complications

Patient	Age	Sex	ASA Class	Surgical procedure	CCI	Specific complications within the first 90 postoperative days
1	77	F	II	Femoral-femoral bypass	36.2	Anemia, Respiratory failure, Hypotension
2	65	M	IV	Total gastrectomy	0.0	None
3	73	F	III	Total abdominal hysterectomy with bilateral salpingo-oophorectomy and lymph node dissection	41.8	Respiratory failure, Hypotension, Sepsis, Hemorrhage requiring transfusion
4	63	F	II	Total thyroidectomy	0.0	None
5	71	M	IV	Low anterior resection	0.0	None
6	82	M	IV	Laparoscopic right hemicolectomy	22.6	Anemia, Acute kidney injury
7	83	M	III	Iliac artery aneurysm repair	29.6	Respiratory failure, Sepsis
8	86	M	III	Left hemicolectomy	0.0	None
9	62	M	III	Partial gastrectomy (Billroth II)	20.9	Respiratory failure
10	71	M	III	Right nephroureterectomy	50.7	Anemia, Sepsis
11	74	M	III	Right lung lobectomy	42.4	Unexpected ICU admission
12	54	M	IV	Carotid endarterectomy	0.0	None
13	55	M	III	Carotid endarterectomy	29.6	Acute kidney injury and hemorrhage at 3rd postoperative day
14	68	M	III	Carotid endarterectomy	20.9	Hypotension at 2nd postoperative day
15	71	F	III	Sigmoidectomy	20.9	Respiratory failure
16	71	M	IV	Radical cystectomy	42.6	Ileus, Sepsis, Urinary infections and pig-tail insertion
17	78	M	IV	Abdominal aortic aneurysm repair	29.6	Sepsis, Anemia and transfusion at 2nd postoperative day
18	79	M	IV	Radical prostatectomy and cystectomy	20.9	Readmission, Sepsis
19	68	M	III	Total gastrectomy	0.0	None
20	82	F	III	Total abdominal hysterectomy with bilateral salpingo-oophorectomy and lymph node dissection	20.9	Sepsis at 2nd postoperative day
21	53	F	II	Low anterior resection	0.0	None
22	68	M	II	Left nephroureterectomy and radical prostatectomy	36.2	Hemorrhage, Acute kidney injury, Rhabdomyolysis
23	66	F	III	Abdominal aortic aneurysm repair	44.2	Sepsis and Anemia at the 7th postoperative day, Abdominal hernia, Unexpected ICU admission
24	66	M	IV	Carotid endarterectomy	66.9	Hypoxia, respiratory infection at the 2nd postoperative day, Acute coronary syndrome, carotid stenosis
25	61	M	II	Abdominal perineal resection	20.9	Respiratory infection

26	74	M	IV	Whipple procedure	47.3	Pulmonary embolism, Sepsis
27	75	M	III	Carotid endarterectomy	0.0	None
28	56	F	II	Right hemicolectomy	0.0	None
29	58	M	III	Laparoscopic right radical nephrectomy	22.6	Hemorraghe, Acute kidney injury
30	61	M	III	Radical nephrectomy and lymph node dissection	95.3	Multiple organ failure (acute kidney injury, liver failure, acute pulmonary edema, ileus, sepsis, myocardial infarction), Unexpected ICU admission
31	74	M	III	Whipple procedure	29.6	Sepsis, Anemia requiring transfusion
32	79	F	III	Mastectomy and lymph node dissection	20.9	Pneumonia
33	62	F	II	Total abdominal hysterectomy with bilateral salpingo-oophorectomy and lymph node dissection	0.0	None
34	24	F	II	Femoral popliteal bypass surgery	0.0	None
35	53	F	IV	Small bowel resection	0.0	None
36	65	F	III	Radical nephrectomy	22.6	Anemia, Sepsis
37	74	M	IV	Carotid endarterectomy	20.9	Respiratory failure at 1st postoperative day
38	74	M	IV	Abdominal aortic aneurysm repair	0.0	None
39	78	F	IV	Partial hepatectomy and cholecystectomy	20.9	Acute kidney injury
40	71	M	IV	Abdominal aortic aneurysm repair	0.0	None
41	67	F	III	Abdominal perineal resection	22.6	Anemia, Sepsis
42	77	M	IV	Total gastrectomy	22.6	Sepsis, Anemia
43	70	M	IV	Sigmoidectomy	0.0	None
44	74	M	IV	Radical prostatectomy and cystectomy	0.0	None
45	71	M	II	Left hemicolectomy	8.7	Anemia
46	52	M	II	Right hemicolectomy	0.0	None
47	68	M	IV	Whipple procedure	0.0	Anemia, Death
48	72	F	III	Small bowel resection	71.7	Anemia, Thrombopenia, Sepsis, Unexpected ICU admission, Several re-operations, Death
49	79	M	IV	Carotid endarterectomy	51.7	Hypotension, Respiratory failure at 2nd postoperative day, Stroke
50	77	F	II	Total abdominal hysterectomy with bilateral salpingo-oophorectomy and lymph node dissection	0.0	Death
51	42	F	III	Total abdominal hysterectomy with bilateral salpingo-oophorectomy and lymph node dissection	20.9	Anemia, Transfusion
52	74	M	IV	Carotid endarterectomy	0.0	None

53	43	M	II	Limb salvage surgery, brachial artery surgery	8.7	Anemia
54	70	M	III	Abdominal aortic aneurysm repair	0.0	None
55	76	M	IV	Abdominal aortic and iliac aneurysm repair	29.6	Sepsis, Respiratory failure
56	69	M	IV	Abdominal aortic aneurysm repair	0.0	
57	77	M	IV	Abdominal aortic aneurysm repair	22.6	Anemia, Sepsis
58	83	M	IV	Abdominal aortic aneurysm repair	8.7	Anemia
59	65	M	IV	Popliteal artery aneurysm repair	29.6	Sepsis, Respiratory failure at 2nd postoperative day
60	77	M	IV	Carotid endarterectomy	42.4	Stroke
61	22	M	III	Retroperitoneal lymph node dissection	8.7	Anemia
62	65	F	III	Right hemicolectomy	0.0	None
63	67	F	III	Left hemicolectomy	20.9	Respiratory infection
64	63	M	III	Laparoscopic right hemicolectomy	0.0	None
65	72	F	IV	Brachial artery surgery	20.9	Acute kidney inury at 3rd postoperative day
66	70	M	III	Abdominal aortic aneurysm repair	8.7	Anemia
67	60	F	III	Mastectomy and lymph node dissection	8.7	Sepsis
68	64	M	IV	Radical prostatectomy	20.9	Sepsis
69	74	M	III	Left hemicolectomy	0.0	None
70	42	F	III	Adrenalectomy	0.0	None
71	64	M	III	Whipple procedure	29.6	Sepsis, Respiratory failure
72	51	M	III	Retroperitoneal lymph node dissection	29.6	Hypotension at 1st postoperative day, Anemia requiring transfusion at 2nd postoperative day
73	75	F	IV	Abdominal aortic aneurysm repair	42.4	Stroke at 3rd postoperative day, Unexpected ICU admission
74	71	M	IV	Aortofemoral bypass surgery	29.6	Hypoxia and anemia requiring transfusion at 2nd postoperative day
75	65	M	IV	Aortofemoral bypass surgery	20.9	Respiratory failure at 1st postoperative day
76	65	M	III	Radical prostatectomy and cystectomy	20.9	Sepsis, Death
77	27	M	II	Right hepatectomy	22.6	Anemia, Respiratory infection
78	76	M	III	Radical cystectomy	59.4	Intestinal rupture, Surgical wound dehiscence, Acute pulmonary edema, Sepsis, Anemia
79	65	F	III	Radical nephrectomy	20.9	Sepsis at 2nd postoperative day
80	89	M	III	Sigmoidectomy	8.7	Anemia
81	47	F	III	Whipple procedure	20.9	Anemia at 3rd postoperative day

82	76	F	III	Right hemicolectomy	29.6	Sepsis, Hemorrhage
83	71	M	IV	Right hepatectomy	20.9	Sepsis
84	62	F	II	Left hemicolectomy	0.0	None
85	66	M	IV	Carotid endarterectomy	0.0	None
86	73	M	IV	Laparoscopic right nephroureterectomy	20.9	Sepsis
87	65	M	II	Partial pancreatectomy	0.0	None
88	52	M	II	Low anterior resection	0.0	None
89	54	M	II	Radical prostatectomy	0.0	None
90	65	F	III	Right hemicolectomy	0.0	None
91	81	F	III	Left hemicolectomy	43.3	Septic Shock, Anemia
92	77	M	III	Whipple procedure	20.9	Respiratory infection
93	81	F	IV	Total abdominal hysterectomy with bilateral salpingo-oophorectomy and lymph node dissection	20.9	Respiratory failure at 2nd postoperative day
94	79	M	IV	Abdominal aortic aneurysm repair	29.6	Sepsis, Transfusion at 2nd postoperative day
95	81	M	IV	Sigmoidectomy	0.0	None
96	85	M	III	Right hemicolectomy	22.6	Sepsis, Anemia
97	44	M	III	Sigmoidectomy	0.0	None
98	61	M	IV	Aortoiliac occlusion	29.6	Sepsis, Respiratory failure at 2nd postoperative day
99	74	M	IV	Abdominal aortic aneurysm repair	8.7	Anemia
100	67	F	IV	Small bowel resection, diaphragmatic hernia	20.9	Respiratory infection
